# The global workforce shortages and the migration of medical professions: the Australian policy response

**DOI:** 10.1186/1743-8462-5-7

**Published:** 2008-05-29

**Authors:** Saxon D Smith

**Affiliations:** 1 19/163 Willoughby Rd, Naremburn 2065, New South Wales, Australia

## Abstract

Medical migration sees the providers of medical services (in particular medical practitioners) moving from one region or country to another. This creates problems for the provision of public health and medical services and poses challenges for laws in the nation state and for laws in the global community.

There exists a global shortage of healthcare professionals. Nation states and health rights movements have been both responsible for, and responsive to, this global community shortage through a variety of health policy, regulation and legislation which directly affects the migration of medical providers. The microcosm responses adopted by individual nation states, such as Australia, to this workforce shortage further impact on the global workforce shortage through active recruitment of overseas-trained healthcare professionals. "Push" and "pull" factors exist which encourage medical migration of healthcare professionals. A nation state's approach to health policy, regulation and legislation dramatically helps to create these "push factors" and "pull factors". A co-ordinated global response is required with individual nation states being cognisant of the impact of their health policy, regulations and legislation on the global community through the medical migration of healthcare professionals.

## Background

We are witnessing a global shortage of medical practitioners [1]. In a microcosm or nation-centric response, developed countries have adopted policies, regulations and legislation which adversely impact upon the greater global workforce shortage. Furthermore, the attempt by these governments to manage the migration of healthcare workers helps underline the difficulties inherent in any attempt to strike the careful balance between of the autonomous rights of individuals to pursue work where they choose with the need to provide increasing diverse medical services to an increasingly demanding general population.

Through a focus on Australia as an example of a developed country this article illustrates that the gripping global shortage of healthcare professionals exacerbates the negative impact of medical migration whereby developed countries strip the dwindling healthcare workforce from the world's developing countries. This complex global problem demands a global response through prudent harmonisation of health law in the pursuit of equitable and just delivery of healthcare and distribution of medical providers. Individual nation states need to be cognisant of their role in harmonisation for equity of health in the global village.

This article debates the ethical and social impact of the current global shortage of medical practitioners and examines Australia's response to this crisis. It is through discussion of the ethical and societal effects of a variety of health policy, regulation and legislation in Australia that the negative global community outcomes that arise from the medical migration can be illustrated. It is ultimately argued that, whilst Australian health policy, regulation and legislation in this area needs to be responsive to the current workforce shortage, it must not in future be responsible for further negative social impacts within the global community.

## Discussion

### The macrocosm – shortage of medical providers in the global community

"The World Health Report 2006: Working together for health" by the World Health Organization (WHO) estimated in 2006, that whilst there are nearly 59.2 million full-time paid health workers worldwide, there is currently a critical shortage of healthcare provider's equivalent to a global deficit approaching 4.3 million health workers [1]. The greatest numerical deficit occurs in South-East Asia, dominated by the needs of Bangladesh, India and Indonesia, while the greatest proportional shortfalls exist in sub-Saharan Africa, where the WHO estimates an increase in health workers of almost 140% is necessary to meet the health needs of its population [1]. Furthermore, the African Region has "24% of the burden (of global disease) but only 3% of health workers commanding less than 1% of world health expenditure" [1]. Conversely, the Americas have "10% of the global burden of disease has 37% of the world's health workers spending more than 50% of the world's health financing"[1].

The large public health discrepancy between developed countries and developing countries is exacerbated by medical migration of healthcare professionals from poor to wealthy regions. In fact close to one quarter (23%) of the current doctor workforce in OECD countries are doctors trained in sub-Saharan Africa, "ranging from as low as 3% in Cameroon to as high as 37% in South Africa" [1]. Several factors exist which encourage medical migration and influence the decision to migrate, including poverty, the search for a better life and livelihood by autonomous individual medical practitioners. Classically it has been argued that "this is provoked by a (growing) discontent or dissatisfaction with existing working/living conditions – so-called push factors, as well as by awareness of the existence of (and desire to find) better jobs elsewhere – so-called pull factors" [1]. However, individual nation states' approach to health policy, regulation and legislation dramatically helps to create these 'push factors' and 'pull factors', such as imperfect labour markets, lack of public funds, bureaucratic red tape and political interference [1]. For example the global lack of funding for medical schools, due to a range of health policy and regulation means in aggregate the world's 1600 medical schools are not producing sufficient numbers of graduates [1]. Furthermore, it has been estimated that Britain's policy of recruiting overseas trained doctors rather than adopting a policy of funding adequate numbers of medical school graduates has saved Britain £65 m in training costs from its recruitment from Ghana alone from 1999–2005 [2]. With insufficient numbers of doctors being trained the chronic shortage of doctors is propagated and the 'brain drain' of health workers migrating from poor to wealthy countries becomes entrenched.

### A microcosm – shortage of medical providers in Australia

Australia is in the grip of a well-publicised healthcare workforce shortage crisis. There are several reasons for these shortages which include "low numbers of medical students being trained along with low numbers entering postgraduate training programs" [3]. The Commonwealth's health monetary and health education policies, regulations and legislation have directly contributed to the current workforce shortages within Australia. Prominent among these were federal government policies in the 1980s[4] and 1990s[5] which limited the number of medical school places in Australia. The federal government's response was based first on the rising costs of Medicare due to the rising average numbers of Medicare services per person[6] and an attempt to balance its fiscal policy [7]. This response was catalysed by an apparent decline in the doctor-to-population ratio from 485:1 to 413:1 over the decade between 1986 and 1996, which helped create a belief that there was not a shortage of doctors, but rather there was uneven urban/rural distribution to match patient needs [6]. Ultimately, the federal government proposed the *Health Insurance Amendment Act *(No2) (Cth) 1996 in response to the perception that "one of the major cost pressures on the health system in Australia is the chronic oversupply of doctors" [7]. This legislation sought to reduce medical school intakes, reduce the numbers of overseas trained doctors migrating, reduce the need to bring in temporary overseas doctors to staff hospitals and, finally, getting a better distribution of doctors between rural and urban regions [7]. Whilst on the one hand there existed a belief that there was an oversupply of doctors, there was a converse shortage of doctors willing to work in the public hospital system. As a result, this legislation responded to the perceived ease for medical graduates to "hang up a shingle and bill Medicare" [7]. The government sought to curb this by limiting the number of medical school places, down from 1500 in 1980s to 1200[8], and by requiring medical graduates to complete postgraduate education in order to gain access to Medicare benefits. It was believed that the 200 Australian students and interns per year would miss out on provider numbers [9]. These medical graduates would "be absorbed by the public hospital system and, in doing so, to begin to ease the onerous workload currently expected of doctors in public hospitals" [7]. This would in turn decrease the need for recruitment of overseas-trained doctors to fill public hospital positions. Furthermore, the legislation was an attempt to control medical migration through the disincentive for them to migrate, requiring them to work for ten years in the public system before they were to gain a medicare provider number [9]. By the late 1990s, there was a dramatic decrease in the growth rate of full-time equivalent GP's billing medicare, down from 4–5% per year in the early 1980s to 0.5% in 1998–1999 [10]. This outcome was justified at the time in the context of cost to the Commonwealth budget for health, the problem was that demand for health care services continued to demand [11]. In fact, until recently, there has been no significant increase in medical school place numbers despite the increasing overall population and increasing healthcare demands from the aging population. This has been most dramatically seen in Queensland where the population has almost doubled over the last 30 years, but the number of places at the University of Queensland medical school has remained static at approximately 225 [12]. In turn, the resultant deficit in local medical graduate numbers impacts on Australia's continued reliance on overseas trained medical professionals to immigrate and fill the shortages in the Australian health care system. As a result Australia's reliance upon overseas-trained doctors is unlikely to diminish in the foreseeable future.

Medical practitioner autonomy has also contributed to the shortage with:

(the) newer generations of doctors differed from previous ones in their emphasis on family and lifestyle issues, and that this affected recruitment and retention. Many newer trainees and resident medical staff were less willing to work the long hours of their predecessors and to accept unpaid overtime. The increasing number of female medical graduates is one factor which had prompted increased demand for part-time work and traineeships and the ability to move in and out of the workforce easily... Lifestyle factors and working hours were also seen as influencing choice of training programs leading to shortages in some areas.[3]

This illustrates the desire of medical practitioners to obtain greater control, self determination and satisfaction in their personal lives. This exercised autonomous desire reflects the multi-factorial impact of doctors graduating later, the increased HECS debt from studying, family circumstances which make them less inclined to work in the public sector [12]. This commitment, as a group, to working shorter hours for lifestyle reasons results in reducing the effective full-time hours that practitioners work.

Other factors that are reducing the supply of "effective full-time practitioners" include: medical practitioner's working hours fell from 48.3 hours per week in 1995 to 44.4 by 2003[13]; retirement rates amongst the "baby boomer" generation of doctors is another likely downward pressure on medical workforce supply[14]; increasing requirement to perform non-clinical duties, e.g. practice management, administration, continuing education [13].

### Australia's response to its shortage of doctors

The short term response has been for state and federal governments to adopt policy, regulations and legislation which enables and encourages active recruitment of overseas-trained doctors. While all Australian states are suffering similar shortages of doctors with the number of local graduates in sufficient to satisfy the growing needs[12], it has become increasingly difficult to recruit medical practitioners from countries with comparable medical systems, such as New Zealand, Britain and Ireland, because these countries are also experiencing workforce shortages with the impact of global medical practitioner migration and autonomy [12]. In consequence the state governments have become highly dependent on recruiting doctors from developing countries. By 2003, nearly 50% of Queensland's Resident Medical Officers were overseas-trained doctors [12]. The percentage of medical graduates from the United Kingdom and Ireland on overseas-trained doctor temporary working visas had fallen from 70% in 1997–1998 to 43% by 2002–2003 [4]. Conversely, the proportion of overseas trained doctors originating from India, Pakistan, Sri Lanka, Malaysia, the Philippines, Bangladesh and 'other' increased from 9.6% to 37.3% [4].

State governments have help break down immigration barriers to enable the active recruitment of overseas-trained doctors by the establishment of medical resource policy incorporating 'Area of Need' provisions. An 'Area of Need' is any position/location (both public and private sectors) in which there is a lack of specific medical practitioners or where there are medical positions that remain unfilled even after recruitment efforts have taken place over a period of time [15]. This policy facilitates the active requirement of overseas-trained doctors to fill this "Area of Need". In fact, In NSW most overseas trained doctors are required to work in an Area of Need when they first come to Australia [15]. Furthermore, the states use existing legislation to enable the recruited overseas-trained doctor to gain medical registration for the purpose of filling an unmet "Area of Need" such as *The Medical Practice Act *1992 (NSW) s7(1)D and *Medical Practitioners Registration Act *2001 (QLD) s135. There also exists the *Mutual Recognition Act *1992 (Cth) and its state equivalents, such as *Mutual Recognition Act *1992 (NSW), which recognises registration between all states and territories in Australia. Thus when an overseas-trained doctors gains registration in one state it allows migration access to all states and territories. The existence of these provisions allows for the targeted recruitment of overseas-trained doctors.

The health law policy of active recruitment of overseas trained doctors is further evidenced by the creation of dedicated recruitment divisions in State health departments to facilitate medical migration. In fact, the Queensland Health Systems Review 2005 specifically recommended the formation of such a department in Queensland Health called RAPTS (Recruitment, Assessment, Placement, Training, and Support For International Medical Graduates) [16]. One of its particular mandated projects is a coordinated marketing and advertising strategy to attract medical graduates, including overseas-trained doctors, for employment in Queensland [16].

The federal government has further contributed to breaking down barriers to immigration barriers and adopted an active recruitment strategy to encourage medical migration of appropriately qualified overseas-trained doctors as the primary short-term solution to address the nation's medical practitioner shortage. This increased recruitment response has been supported by "a range of measures to simplify the process for overseas trained doctors entering and working in Australia" with government amending immigration policy to increase the duration of years for temporary visas for overseas-trained doctors from 2 years to 4 years [17]. Further changes to immigration policy by the government in May 2004 saw medical practitioners included on the Department of Immigration's "Skilled Occupations List" which means overseas-trained doctors no longer need a sponsor to immigrate [17]. As of March 2006 medical practitioners were included on the Department of Immigration's Migration Occupations in Demand List [18]. If an occupation is on the nominated Migration Occupations in Demand List, the visa application will receive priority processing [18]. Furthermore, The *Migration Act *1958 (Cth) s30 now allows for temporary or permanent migration visas. There are three designations for entry to Australian medical practice for overseas-trained doctors under temporary residency visas:

1) Temporary Business (long stay) visa 457[19], which allows an employer to sponsor the doctor for up too 4 years with option of renewal.

2) Medical practitioner visa 422[20], which requires the sponsoring employer to demonstrate that the position is an identified "area of need" and they have been unable to recruit an Australian resident to fill the position. It allows sponsorship for 4 year visa with option of renewal.

3) Occupational Trainee visa 442[21], which requires appointment as junior doctor and as trainee specialist in hospitals, and Department of Immigration receipt of a letter from relevant medical college stating that a training program has been prepared and will be carried out in the hospital. Importantly the holder of this visa does not have to work in an "area of need". It is valid for twelve months with option to renew.

Permanent residency and thus entry to medical practice either can be obtained through successful completion of the Australian Medical Council examinations, or through employment as provisionally registered junior hospital doctor, or as General Practitioner in "area of need" locations. There are four main permanent resident visa categories: Employer nomination scheme; Regional sponsored migration scheme; Labour Agreements; and, General Skilled migration [22]. The federal government has adjusted their immigration policy in response to the doctor shortage in Australia and established multiple permanent and temporary residency visa categories available to overseas-trained doctors. This facilitates active recruitment of overseas-trained doctors by removal of immigration barriers. According to the Australian Government Department of Health and Ageing these new immigration arrangements:

"...have been established to improve the processes for attracting appropriately qualified overseas trained doctors to the Australian medical workforce. During 2003–04, a total of 1,895 overseas trained doctors were approved... in comparison with 1,501 approved during the 2002–03 year. This represents an increase of 26 per cent."[17]

Furthermore, the Commonwealth Department of Health and Ageing has developed a website called 'DoctorConnect', which provides information aimed at overseas trained doctors, employers and those advising them [23].

Another example of the commonwealth government's preference to actively encourage migration to solve Australia's medical workforce shortage, was seen in 2002 when it amended immigration policy to allow international students who were trained at Australian medical schools as full-fee paying students, to be eligible for placement as interns at public hospitals for a one year period only [17]. However, by 2003 this short-term solution was extended to an indefinite time frame as well as allowing these students to apply for permanent residency and thus access vocational specialist training programs [17]. As a result, in 2004, nearly 135 of these students had accepted intern placements in public hospital positions [17]. It has only been in the last two years that the federal government has finally responded to the shortage of doctors by increasing the number of government funded medical school places nationally with the number of Australian medical graduates set to increase by 60% by 2010 [13].

This most recent response by government to increase the number of medical graduates produced by Australia potentially creates its own inherent difficulties and may result in Australia's continued reliance on actively encouraging medical migration. First, there is a lack of medical educators and supervisors within Australia. This is a direct symptom of the general numerical deficit in medical professionals where the need to fulfil service delivery demand results in less time available to fill medical education roles. Therefore in order to satisfy the demand for medical educators a program of active recruitment of overseas-trained medical academics may have to be pursued in the coming years. Thus it could be argued that rather than an increase in medical school graduate numbers acting to curtail the demand for medical migration, it may in fact increase the need for active recruitment of overseas-trained doctors. Second, there exist significant flow on effects down the chain of the continuing education and post-graduate specialty training. Seemingly there has been a lack of planning with respect to funding, availability and supervision for specialty training positions should this influx of medical graduate desire to pursue post-graduate specialty training. It may be argued that novel training environments such as training within the private hospital system could help to provide a partial solution to this particular flow on effect. However, this may potentially result in the need to extend the already long process of specialty training so that trainees obtain the requisite training experience to satisfy the level of skill and knowledge for admission to their chosen specialty. Another potential solution would be to provide early streaming of medical students into specialty areas whilst still completing their medical degree. This approach is to be trialled by the New South Wales Department of Health which have negotiated early streaming of medical students at the University of New South Wales into radiology and pathology [24]. Alternatively, it could be argued that such an increase in training may not be necessary should it become acceptable to have trainees initially qualifying with a lower standard of generalist knowledge. This could result in the super-subspecialist who specialises in a narrow stream of expertise within the current defined clinical specialties. Again this flow-on effect may not supply enough appropriately trained medical professionals to satisfy the increasing demand for particular medical services, and thus Australia would remain reliant on overseas-trained doctors to fill the clinical holes in our health care system.

### Global impact of Australia's health law policy changes

Australia's active recruitment policy of overseas-trained doctors has an ethical impact on the global workforce shortage as well as potential negative impact upon the delivery of healthcare within Australia's public health system. As an individual nation state, Australia's recruitment of overseas-trained doctors from developing countries is a detriment to the source country where their skills are most needed.

### Ethical global impact

Since 1997, medical migration has seen the proportion of overseas trained doctors originating from India, Pakistan, Sri Lanka, Malaysia, the Philippines, and Bangladesh dramatically increase [4]. According to the WHO these are some of the countries with critical shortages of medical practitioners [1]. The health services of these countries suffer adverse health outcomes from the "loss of trained personnel, leading to diminished health care, reduced talent in the system, diminished management and supervision, and a higher unmanaged disease burden" [25]. When a country has a fragile health system, these losses from its workforce can bring the whole system close to collapse and the consequences can be measured in lives lost [1].

When large numbers of doctors migrate, the countries that financed their education lose a return on their investment and unwillingly provide the wealthy countries to which their health personnel have migrated, with a kind of "perverse subsidy" [1]. Also by allowing Australian university-trained international medical graduates placements in public hospitals and access to post-graduate training programs, raises the issue whether the government should have made available those same places to Australian students. By relying on international full-fee paying students to help fund/finance the university training and then relying on them to remain, Australia is also denying the home countries of these medical graduates.

However, the migration of medical practitioners across international borders can have beneficial effects for their former source country. If doctors do return home to the former source country, they return with significant skills and expertise that they have acquired. However, in the event that medical practitioners do not return to the former source country the billions of dollars in remittance can have a beneficial impact with influx of money sent home by migrants, which has been "associated with a decline in poverty in low income countries" [1]. In 2001, it was estimated that US$72.3 billion dollars of remittance was sent "home" by emigrants and is the second-largest source of external funds for developing countries [26]. Even though medical migration contributes significantly to this figure, the strained healthcare systems in the source country from which doctors have migrated, do not necessarily benefit from re-investment of these funds.

### Service delivery impact

The delivery of healthcare in Australia is also directly impacted by the recruitment of overseas-trained doctors. Simplistically one would consider an increase of service providers to equate with an increase in service delivery, however, this can also result in a detrimental impact on the health of Australians, as has been shown in recent adverse outcome events, such as malpractices exposed by Queensland Public Hospital Commission of Inquiry 2006. There are differing levels of knowledge and skills among overseas-trained doctors because medical education programs vary widely, in terms of duration, curriculum, standards and quality and evaluation, from country to country [27]. Although this does not necessarily equate with inferior medical training which overseas-trained doctors receive, it is very difficult to compare medical schools globally as they are a heterogeneous group and have widely different origins, backgrounds, training and capabilities. Inexperience with or lack exposure to the level of technology used routinely in Australian hospitals, as well as unfamiliarity with commonly encountered Australian health problems, contribute to the clinical challenges faced by over-seas trained doctors [27]. Also significantly, English language difficulties create potential barriers between overseas-trained doctors, their colleagues and their patients and many overseas-trained doctors have received little or no formal training in communication skills [27]. This can negatively impact the delivery of healthcare by impairing the doctor-patient relationship because of poor communication skills [27]. In fact, substantial research into doctor-patient communication indicates that poor communication skills contribute to problems in history-taking, diagnosis, management, and provision of information to the patient [27]. Until May 2004, the Medical Board of Queensland did not have a policy of ensuring that overseas-trained doctors seeking registration could speak English proficiently [12]. Furthermore, overseas-trained doctors are confronted by a range of other cross-cultural challenges including sex-role differences, discrimination and change in status [27].

While, the federal and state government's current policy to increase medical student places at Australian Universities is an appropriate response to address the overall workforce shortage and decrease Australia's reliance on recruitment of overseas-trained doctors, it is not without its own potential problems. A cautionary counterargument has been proposed which suggests that the current "shift" into a boom growth phase in Australian medical practitioner supply will create a "square wave shift" with a large infrequent adjustment in supply increasing the risk of overcorrection [13]. This overcorrection could result in more medical graduates than available jobs, especially GP/speciality training positions and if this were to happen Australia may "shift" from being an inbound country for medical migrants to being an outbound country, suffering the potential effects of 'brain drain', with its medical graduates seeking employment opportunities in the global community.

### A positive future direction to address medical migration

As a result of globalisation, nation states no longer operate in a relative political and policy isolation and whilst Australia looks to solve its own workforce shortages, it also needs to be aware that their policies may be having dire consequences elsewhere. The removal of immigration barriers and the breakdown of international boundaries through globalisation make it difficult to legislate or regulate the autonomous individual's decision to migrate for their own ends. The key challenge is to "create socially and environmentally forms of globalisation that provide the greatest benefit and least cost"[28] to the global community. The complicated cascade effect of medical migration does require a co-ordinated global response. There exists a complex interplay between autonomous individual doctors, their workplace and market forces which reflect the emotionally and politically charged contexts in which medical migration operates. A global policy to minimise the inequalities between nation states, diminish the negative impacts of medical migration and respect individual autonomy requires action, and requires "receiving" countries to be cognisant of the impact their healthcare policies have on the global village, especially with respect to source countries. It has been argued that receiving countries should be aware of and concerned with the adverse consequences of active recruitment through enabling policies which encourage medical migration. A growing cognisance of the negative impact of medical migration on source countries may be remedied by adoption of more appropriate health policies by "receiving" countries. At the heart of it, promoting equity within a country and between countries is consistent with the Australian value of a 'fair go' [29]. However, aspects of Commonwealth and State Health and fiscal policy, regulations and legislation need to be addressed in order to address the underlying causes of the growing reliance on overseas-trained doctors and alleviate the need for active recruitment programs.

### Strategies for a positive national response

Good national policy can decrease the reliance on overseas-trained doctors, and therefore decrease the need to actively recruit from the small global pool to the detriment of developing nations.

First, the Commonwealth should adopt a proactive policy of appropriate numbers of medical school places. Scott et al contest that the better management of human resources in a developed country like Australia would result in a fewer areas of need and thus diminish the need to actively recruit overseas trained medical professionals [30]. In spite of this, there has been two decades of essentially stagnant number of Commonwealth funded medical school places. This perpetuating deficit has significantly contributed to the reliance on medical migration as a permanent solution to Australia's workforce crisis. In late recognition of this the Commonwealth has commenced a policy of increasing the number of funded medical school places with 2100 graduating places by 2010. Unfortunately, this has been a reactive policy response to a significant deficit that has developed over two decades neglect, rather than the development of proactive sustainable policy by government and may result in increased demand for overseas trained doctors to fill medical education roles or generalist service delivery roles. If Australia were to pursue a policy of appropriate medical school places at university, it could mean that there would be a more adequate supply of human resources within our health care system and decrease the need to actively recruit overseas-trained medical graduates.

Second, Australia should review its recent approach to skilled working visas. As discussed in detail earlier Australia has demonstrated a willingness to change visa requirements for medical professionals which has enabled the active recruitment of overseas-trained doctors. However, legislation or policy could for example be drafted to amend to the *Migration Act *1958 (Cth) to fix the number of the visas available in each of the various categories available to overseas-trained doctors; numerical restrictions may include specific maximal quotas allowed from countries, with particular emphasis on developing countries; creative mutual obligation contracts between nation states; skill recirculation; mutual support and interdependence for health infrastructure between nation states [26]. Furthermore, Australia is well positioned to issue non-extendable visas for overseas-trained doctors. Whilst this may still result in a short term migration of overseas-trained medical professionals, it would in the medium to long term see the same medical professionals return to their home countries in a defined timeframe and with widened professional skills.

Third, some argue that skilled health professionals could be seen as exportable assets with the receiving country paying compensation to the source countries for their loss of trained personnel [30]. However this approach lacks ground in good ethics because it does not actually address the practice of active recruitment of medical professionals from developing countries. Whilst there may be a financial compensation there is no guarantee that this incoming money where to be directed into the health care of the source country. Furthermore, even if the money was directed appropriately, for example by some multi-lateral agreement, the decreased number of health care professionals available in the 'source' country potentially would result in the purchase of unusable equipment or stock piles of vaccines or medicines that are unable to be administered. For example, this could result in no one to staff the clinic which provides the vaccine that the compensation money bought, or lack of trained people to run the MRI machine that was purchased. This would be the same outcome should an 'in-kind' approach be taken, such as provision of medicines or medical equipment rather than direct financial compensation. Alternatively, it has been suggested that recipient countries could invest in enhancing training and skills development in the countries exporting skilled staff [30]. However, this approach again does not address the fact that there is stripping of medical professionals from sparsely resourced developing countries. As illustrated by the £65 million the UK government saved by recruiting medical professionals from Ghana from between 1999–2005[2] earlier developed countries receive significant cost savings by recruiting trained healthcare professionals from other countries rather than training them themselves. However, these financial and social capital based approaches need to be considered as a disincentive to developed countries and not an incentive for developing countries for them to be effective. The disincentive should be set at a level where it is more advantageous for the developed nation to address their own internal deficits directly rather than through a program of active recruitment of overseas-trained doctors. Furthermore, this would then guard against the compensation being an incentive for developing countries to encourage their medical professional to migrate and thus the same end result of stripping developing countries of these limited resources.

Any response by a developed country, such as Australia, requires a concerted long-term commitment to improve the training and education systems in their own countries. Whilst some argue that there exist solutions at a national level such as compensation for developing countries, these are fraught with potential abuse and all too often would appear band-aid solutions. Ultimately a positive national response requires an ethical responsibility of developed countries to manage their own human resources appropriately, such as ensuring a policy of adequate medical graduate and revising current visas classes for overseas trained medical professionals.

### Strategies for a positive global response

Within the context of globalisation it is not possible to control medical migration resulting from autonomous individual decision making on the part of the healthcare professional. However a global framework is required to view the actions of countries, like Australia, who continue to pursue programs of active recruitment to solve nation state workforce shortage their actions must be viewed within a global framework.

First, the adoption of a recruitment code of ethics may help to modify or moderate the practice of active recruitment behaviour. The Department of Health in The United Kingdom has led the way developing its first Code of Practice for International Recruitment in 2001 which has been updated 2004 [31]. The aim of the code is for developing countries not to be targeted for recruitment of healthcare personnel unless the government of that country formally agrees via the Department of Health (UK). Furthermore, the Commonwealth Secretariat in 2002 also developed a Commonwealth Code of Practice for International Recruitment of health workers [32]. However, there are concerns that such voluntary codes are only a 'quick and cheap' strategy to change employment behaviour and the successful implementation of a code of practice requires health laws for internal monitoring, external monitoring and incentives/sanctions. However, the experiences of environmental lobbyists such as Greenpeace and those concerned with working conditions in "sweatshops" in developing countries raise questions about the effectiveness of these instruments. Furthermore establishing and implementing these instruments on a national scale is a major undertaking requiring substantial systems development and inevitably results in some expense. However, they do act as a statement of governmental policy against active recruitment programs, especially ones targeting developing countries. Unfortunately, at present, Australia does not have a Code of Ethics governing recruitment of international healthcare professionals, but rather an active recruitment approach with seemingly little consideration for the ramifications to the source countries and the global impact.

Second, developed countries could pay compensation, either financial or social capital, to developing countries from which medical professionals are recruited could be implemented. However, as mentioned above in the section examining a national strategy, a compensation based solution has several inherent problems with appropriateness and would necessitate a disincentive styled approach. Furthermore, it would require a high level of international cooperation between countries with inherent competition with fiscal and ethical agendas which would make harmonisation difficult to achieve. Scott et al suggest that this approach would require oversight by an international body such as WHO and have its authority based within international law [30]. However, as with any international law approach, the individual nation state's legislation may place it in conflict with any international treaty proposed. Perhaps a key mechanism would be an attempt to harmonise health law pertaining to medical providers for the global community by the establishment of a centralised power for health law, such as a development of World Health Organization or World Trade Organization. The internationalisation of Public Health could be enabled by the networks of technical expertise among public health officials and agencies exist and operate within international organisations (for example PAHO, WHO) and under regional diplomatic platforms (for example APEC).

## Conclusion

As enunciated by the late Lee Jong-wook, Director General of World Health Organization:

There is a chronic shortage of well-trained health workers. The shortage is global, but most acutely felt in the countries that need them most. For a variety of reasons, such as the migration... of health workers, countries are unable to educate and sustain the health workforce that would improve people's chances of survival and their well-being.[1]

Although the crisis has the potential to worsen over the coming years as demands for service providers escalate markedly in all countries – rich and poor and the deep differences between parts of the world seem to preclude the achievement of complete consensus, the problems of globalisation require the pursuit of a greater harmony between states within the global village.

Australia is well positioned as a global participant to play an exemplary role by pursuing ethical policies relating to its own recruitment approaches. Furthermore, Australia could contribute to the development of a global response to the migration of medical professionals to maximise the benefits and minimise the harm for all global citizens.

## Competing interests

The authors declare that they have no competing interests.

## Authors' contributions

SS carried out all the research, drafted the manuscript and approved the final manuscript.
